# Editorial: The association between viral infection and human cancers

**DOI:** 10.3389/fmicb.2024.1371581

**Published:** 2024-02-02

**Authors:** Ming Hu, Bin Wang, Jinlin Li, Chengjun Wu

**Affiliations:** ^1^Department of Special Medicine, School of Basic Medicine, Qingdao University, Qingdao, Shandong, China; ^2^Department of Medical Biochemistry and Microbiology, The Biomedical Center, University of Uppsala, Uppsala, Sweden; ^3^School of Biomedical Engineering, Dalian University of Technology, Dalian, China

**Keywords:** viral infection, cancers, prognosis, therapy, oncolytic virus

The World Health Organization estimates that 15.4% of all cancers are attributable to infections and 9.9% are linked to viruses (Plummer et al., 2016). Cancers that are attributable to infections have a greater incidence than any individual type of cancer worldwide. Eleven pathogens have been classified as carcinogenic agents in humans by the International Agency for Research on Cancer (IARC; Bouvard et al., 2009). After *Helicobacter pylori*, the four most prominent infection-related causes of cancer are estimated to be viral: human papilloma virus (HPV), hepatitis B virus (HBV), hepatitis C virus (HCV), and Epstein–Barr virus (EBV; Zapatka et al., 2020). They are all DNA viruses, except for HCV. Viral infection can lead to uncontrolled cell proliferation and transformation through interfering with cellular regulatory proteins, inactivating tumor suppressor genes, evading host immune responses, inducing persistent inflammatory reactions, causing epigenetic changes, stimulating angiogenesis and activating telomerase (Chu et al.).

The persistent high-risk HPV (HR-HPV) infection has been strongly associated with several types of cancer, such as cervical cancer (99.7%), head and neck squamous cell carcinomas (60%), anal cancer (93%), vulvar cancer (69%), vaginal cancer (75%), and penile cancers (47%) (Brakebill et al., 2023; Liu and Wallace, 2023; Pisani and Cenci, 2024). Lin et al. reported a significantly higher risk of breast cancer in HPV patients than in non-HPV patients, with an adjusted hazard ratio of 2.271 in Taiwan. The mechanism by which HPV causes cervical cancer is relatively well-defined. The rodent and human cells have been shown to undergo immortalization and transformation upon sustained expression of E6 and E7 proteins translated from HPV early genes in cell lines (Lou et al., 2022). An article by Chu et al. reports the successful construction of a recombinant virus that expresses HPV16 E7 protein in cervical cancer cells and induces the up regulation of CD36 gene, which is involved in HPV-related oncogenic pathways. This also provides a potential platform for developing replicative HPV recombinant vaccines. On the correlation between the prognosis of HPV infection and lesion recurrence, Lu et al. conducted a retrospective study after cervical conization in 300 patients. They found that the HPV-negative rates increased over time after surgery. Also, patients with HPV type 16 infection had the highest risk of cervical squamous intraepithelial lesions.

Another group of DNA viruses closely associated with tumors are members of the family *Herpesviridae*. Lee et al. have found that Kaposi's sarcoma-associated human herpes virus (KSHV) infection can induce high-mobility group box 1 (HMGB1) to transfer from the nucleus of endothelial cells to the extracellular space and secrete into the culture medium. HMGB1 plays a key role in the pathogenesis of Kaposi's sarcoma (KS) by regulating the secretion of cytokines and growth factors, affecting the tumor microenvironment. Although HCMV is not a clearly defined oncogenic virus, numerous reports in recent years have shown its correlation with various types of tumors (Hu et al., 2021, 2022). In tumor cells, HCMV may hijack the mRNA nuclear export machinery, thereby changing the translation of cellular genes and promoting tumor progression (Li et al.).

An oncolytic virus can be either a naturally occurring DNA virus, or a genetically engineered virus that selectively infects and kills cancer cells, while sparing normal cells. It can also stimulate the immune system to attack the tumor. Hao et al. provides insights into the molecular mechanisms of EV-A71 oncolysis and its potential anti-tumor efficacy in glioma. They identified *PTBP1*, a gene that is downregulated by EV-A71 infection in glioma cells, as a potential prognostic biomarker and therapeutic target for glioma1.

The research articles included in this Research Topic investigated the cellular transformation mechanisms employed by DNA tumor viruses and also shed light on novel therapeutic targets, diagnostic tools, and treatment strategies that can be implemented in clinical settings to effectively treat tumors caused by oncogenic viruses. In conclusion, these articles clearly develop a better understanding of the underlying molecular processes involved in viral-induced tumorigenesis.

## Author contributions

MH: Conceptualization, Writing—original draft, Writing—review & editing. BW: Conceptualization, Writing—review & editing. JL: Conceptualization, Writing—review & editing. CW: Conceptualization, Writing—review & editing.
